# Neonatal and Maternal Risk Factors for Indirect Hyperbilirubinemia: A Cross-Sectional Study from Bahrain

**DOI:** 10.1155/2022/5199423

**Published:** 2022-09-09

**Authors:** Hasan M. Isa, Noor Y. AlBuainain, Fatema Y. Bunajem, Abdulrahman S. Masood, Yusuf A. Bucheery

**Affiliations:** ^1^Pediatric Department, Salmaniya Medical Complex, Manama, Bahrain; ^2^Pediatric Department, Arabian Gulf University, Manama, Bahrain; ^3^Pediatrics Department, King Hamad University Hospital, Muharraq, Bahrain; ^4^Salmaniya Medical Complex, Manama, Bahrain; ^5^Mohammed Bin Khalifa Bin Salman Al Khalifa Specialist Cardiac Center, Riffa, Bahrain

## Abstract

**Results:**

Out of 555 records, 404 neonates were included. Among those, 209 (51%) were males and 275 (68.1%) were Bahraini. The median indirect bilirubin level at presentation was 218 (interquartile range, 174-270) *μ*mol/L. ABO incompatibility was the commonest risk factor for neonatal indirect hyperbilirubinemia (*n* = 152, 37.6%) followed by glucose-6-phosphate dehydrogenase (G6PD) deficiency (*n* = 130/400, 32.5%). Age (>25 years) was the commonest maternal risk factor (*n* = 331, 81.9%) followed by cesarean delivery (*n* = 137, 33.9%). Neonates with ABO incompatibility had a significantly higher mean indirect bilirubin level compared to those with other risk factors (234.9 ± 68.5 versus 225 ± 82.2 mmol/L, respectively) (*P* = 0.04). Phototherapy use significantly increased along with the rise of bilirubin level (*P* < 0.0001). Intravenous immunoglobulins (IVIG) and exchange transfusion were used in 44 (10.9%) and 14 (3.5%) patients, respectively. Neonates who received IVIG had significantly higher bilirubin levels than those who did not (*P* = 0.005). Male newborns (*P* = 0.008), Bahrainis (*P* = 0.001), those with reticulocytosis (*P* = 0.001), and those who received IVIG (*P* = 0.001) were more prone to have associated risk factors.

**Conclusion:**

ABO incompatibility, G6PD deficiency, and older maternal age were the commonest neonatal and maternal risk factors for developing neonatal indirect hyperbilirubinemia. Bahraini, male newborns, reticulocytosis, and IVIG use were associated with these factors. Early detection of such factors through screening can aid in immediate management to prevent serious complications of this common condition.

## 1. Introduction

Neonatal jaundice is the clinical manifestation of hyperbilirubinemia [1]. It is the most common cause of admission in neonates worldwide [2]. It is defined as the yellowish discoloration of the sclera, skin, and mucus membranes due to bilirubin deposition. It occurs mainly during the first week of life affecting almost 60% and 80% of term and preterm neonates, respectively [2]. It occurs when the total serum bilirubin levels exceeded 5 mg/dL [1, 3].

Bilirubin is a byproduct of red blood cell (RBC) destruction. Initially, hemoglobin is released; then, it gets broken down in the spleen, liver, and bone marrow to form unconjugated bilirubin [4]. Unconjugated bilirubin binds to albumin to be transported in the circulation to the liver, where it combines with glucuronic acid to produce the more water-soluble form, the conjugated bilirubin [4]. Disruptions in any step in this pathway can lead to unconjugated, conjugated, or mixed hyperbilirubinemia. The management of hyperbilirubinemia is essential to prevent complications such as recurrence, kernicterus, and even death [5, 6].

Neonatal jaundice can be subdivided into physiological and pathological jaundice. Physiological jaundice is a condition in which unconjugated hyperbilirubinemia is observed. It usually resolves without any serious complications. However, pathological jaundice is more critical. It can be due to unconjugated or conjugated hyperbilirubinemia. It is considered in cases of breastfeeding jaundice, breast milk jaundice, hemolytic jaundice (Rhesus factor (Rh) incompatibility, blood group (ABO) incompatibility, and glucose-6-phosphate dehydrogenase (G6PD) deficiency), hypothyroidism, urinary tract infection (UTI), sepsis, TORCH infections, and cephalohematoma [5, 7–10].

Neonatal hyperbilirubinemia risk factors include low birth weight, O blood group in mothers, Rh-negative mothers, sepsis, infants of diabetic mothers, prematurity, failed lactation in exclusively breast-fed infants, and a family history of neonatal jaundice [2].

Several treatment options were implicated for the management of unconjugated hyperbilirubinemia. The initial step in the management is phototherapy. If hyperbilirubinemia is resistant or severe, exchange transfusion (ET) could be considered. ET eliminates bilirubin and possibly antibodies from the blood in suspected cases of Rh isoimmunization or ABO incompatibility. Less frequently used treatment options are phenobarbitone, intravenous immunoglobulin (IVIG), and metalloporphyrin [2, 5].

Since studies about risk factors of neonatal indirect hyperbilirubinemia in Bahrain and Arabian Gulf region are scarce, this study is aimed at identifying the most common neonatal and maternal risk factors of indirect hyperbilirubinemia and at comparing neonates with risk factors and those without in terms of clinical and laboratory characteristics, to provide an updated data about this condition.

## 2. Materials and Methods

### 2.1. Patients and Materials

This study is a cross-sectional retrospective study. Data was collected and reviewed through electronic and printed medical records of all neonates presented to the Pediatric Department at Salmaniya Medical Complex, Kingdom of Bahrain, with a clinical impression of neonatal jaundice from January to December 2020. Initially, 555 medical records were reviewed. Duplicate registries of those who were readmitted for indirect hyperbilirubinemia management (*n* = 54) were removed, and they were included in the total data only once of the first presentation. Patients with insufficient data (*n* = 96) were also removed as a first step.

### 2.2. Inclusion Criteria

All neonates admitted for evaluation, investigation, and management of neonatal indirect hyperbilirubinemia were included.

### 2.3. Exclusion Criteria

Patients were excluded from the study if they presented to the hospital with neonatal jaundice but were not admitted and if they were diagnosed with direct hyperbilirubinemia.

### 2.4. Data Collection

Maternal risk factors of neonatal indirect hyperbilirubinemia including maternal age if >25 years, maternal race if East Asian, gestational diabetes (GDM) or diabetes mellitus (DM), urinary tract infection (UTI) in mothers, hypothyroidism, hyperthyroidism, and the mode of delivery were collected. Neonatal demographic data including sex, nationality, age at presentation, gestational age, birth weight, feeding type, management, and length of hospital stay were also gathered.

Laboratory evaluations include complete blood count (CBC) with differentials, reticulocyte count, maternal and newborn blood group and Rh factor, liver function test (LFT), total serum bilirubin (TSB) and indirect serum bilirubin (ISB) levels at admission, direct Coombs test (DCT), serum free thyroxine (T4) and thyroid-stimulating hormone (TSH) levels, blood and urine cultures, TORCH screen, erythrocyte G6PD status (deficiency if <650 U/L in males or <400 U/L in females), and hemoglobin electrophoresis.

The need for phototherapy and double-volume whole-blood ET, including the duration of phototherapy, was also included. Kernicterus was suspected in patients who had early neurological symptoms and signs. Radiological imaging such as skull ultrasound, computed tomography scans, and magnetic resonance imaging of the brain was reviewed for those patients.

### 2.5. Ethical Consideration

This study was conducted in agreement with the Helsinki Declaration and was approved by the Research and Research Ethics Committee for Government hospitals, Salmaniya Medical Complex, Bahrain (IRB number: 5070121). Informed consent was obtained from each infant's parent or legal guardian upon admission.

### 2.6. Statistical Analysis

Data were initially entered in Excel sheet and then transferred to IBM SPSS Statistics program version 21.0 (IBM Co., Armonk, NY, USA) for analysis. Categorical variables were presented as frequencies and percentages. Continuous variables were presented as mean and standard deviation or median and interquartile range (IQR) according to distribution normality. The Kruskal-Wallis test was used to compare different types of phototherapies while the Mann–Whitney *U* test was used to compare neonates who received ET and/or IVIG with those who did not in terms of the mean indirect bilirubin level. To study the possible risk factors for hyperbilirubinemia, patients were divided into two groups, neonates with risk factors (one or more) and those without. Both groups were compared using Fisher's exact test or Pearson's chi-square test for categorical variables (sex, nationality, mode of delivery, phototherapy, and IVIG use). Continuous variables (white blood cell count, hemoglobin, hematocrit, platelets, and reticulocyte percentage) were compared using the Student *T* test or Mann–Whitney *U* test. Moreover, to differentiate hemolytic disease of the newborn (HDN), such as ABO incompatibility and/or Rh incompatibility, from other causes, patients were divided into two groups based on whether HDN was present or not. Both groups were compared in terms of the mean serum indirect bilirubin level using the Mann–Whitney *U* test. *P* value < 0.05 was considered statistically significant. Confidence interval was set at 95%.

## 3. Results

During the study period, a total of 405 neonates were presented to our institution with indirect hyperbilirubinemia. One patient was excluded as he was not admitted to the hospital. The remaining 404 neonates (no twins) and their mothers were included. The demographic data of the included patients is shown in Table 1.

Two hundred and nine patients (51.7%) were males. Two hundred and seventy-five (68.1%) patients were Bahraini, while 129 (31.9%) were non-Bahraini (55 were from India; 14 from Pakistan; 11 from Yemen; eight each from Bangladesh and Philippines; seven from Egypt; four from Jordan; three from Iran; two each were from Morocco, Nepal, Sudan, and Sri Lanka; one each was from Bhutan, Ethiopia, Indonesia, Kenya, Oman, Somalia, Syria, Thailand, and Turkey, while two were from other nonspecified countries).

Neonatal and maternal risk factors for indirect hyperbilirubinemia are shown in Table 2.

Of 404 neonates, 315 (78%) had risk factors for neonatal indirect hyperbilirubinemia. ABO incompatibility is the commonest factor and was found in 152 (37.6%) patients, followed by G6PD deficiency which was found in 130 (32.5%) neonates out of the 400 (99%) tested patients. Of the latter, 46.5% (*n* = 126/271) were Bahraini, while only 3.1% (*n* = 4/129) were non-Bahraini (*P* < 0.0001).

Possible maternal risk factors for neonatal indirect hyperbilirubinemia were found in 360 (89.1%) mothers, while 44 (10.9%) mothers had no risk factors. Maternal age (>25 years) was the commonest risk factor and found in 331 (81.9%) mothers, followed by cesarean section delivery, which was found in 137 (33.9%) mothers.

Results of laboratory tests and radiological imaging performed for neonates with indirect hyperbilirubinemia are shown in Table 3.

Complete blood count revealed that one (0.25%) patient was anemic, 27 (6.7%) had thrombocytopenia, 49 (12.1%) had thrombocytosis, two (0.5%) had leukopenia, 254 (62.9%) had leukocytosis, five (1.2%) had reticulocytopenia, and 303 (75%) patients were found to have reticulocytosis.

Three (1.8%) neonates had positive blood cultures. They grew Staphylococcus haemolyticus, Staphylococcus pasteuri, and Staphylococcus warneri (0.6%) in the first patient; Staphylococcus warneri (0.6%) in the second patient; and Staphylococcus epidermidis and Escherichia coli (E. coli) (0.6%) in the third patient. Seven neonates had positive urine cultures (7.7%), E. coli grew in three neonates (3.3%), and Enterococcus faecalis and Klebsiella pneumonia grew in one each (1.1%). Two cultures showed mixed growths, which are most likely contaminated (1.1%).

Maternal urine culture was positive in 27 (10.3%) mothers; it grew Group B streptococcus (GBS) in 12 (4.7%), E. coli in six (2.2%), Klebsiella pneumonia in four (1.5%), Candida albicans in two (0.7%), and Citrobacter koseri (0.4%), Enterococcus aerogenes (0.4%), and Candida albicans with Gardnerella vaginalis in one each (0.4%). Maternal high vaginal swab was positive in 43 (40.2%) mothers. It showed Gardnerella vaginalis in 13 (12.1%), GBS and Candida albicans in ten each (9.3%), and Trichomonas vaginalis in one (1%). The rest showed mixed growth between the previous organisms (9.3%).

Skull ultrasound was done for 35 (8.7%) infants; all were unremarkable except for one which showed grade 2 intraventricular hemorrhage. Abdominal ultrasound showed hepatosplenomegaly and biliary sludge, each in one patient.

Table 4 shows different types of management of neonates with indirect hyperbilirubinemia.

Data about phototherapy use was found in 342 (84.7%) patients, while 62 patients (15.3%) had no data about receiving phototherapy. Out of the latter, three already received IVIG, two received ET, two received both IVIG and ET, one received phenobarbital, and one received antibiotics for the management of UTI. The 53 remaining of the 62 infants had no data regarding phototherapy; however, their indirect bilirubin levels were within phototherapy range, giving the possibility that they received phototherapy, but they were not included in the analysis as data was not documented. The use of phototherapy significantly increased along with the rise of the mean indirect bilirubin level (*P* < 0.0001). Neonates who received ET had higher mean level of indirect bilirubin, but this difference was not statistically significant (*P* = 0.669). Yet, neonates who received IVIG had significantly higher indirect bilirubin levels compared to those who did not (*P* = 0.005).

On comparing newborns with risk factors (one or more) to those without, male (*P* = 0.008), Bahraini (*P* = 0.001) newborns, those with high reticulocytes (*P* = 0.001), and those who received IVIG were more prone to have associated risk factors of neonatal indirect hyperbilirubinemia (Table 5).

However, mode of delivery; other laboratory investigations such as white blood cell count, hematocrit, hemoglobin level, platelets, total bilirubin, and indirect bilirubin; phototherapy use; and ET were not associated with the presence of neonatal indirect hyperbilirubinemia risk factors.

On comparing neonates with HDN (Rh and ABO incompatibility combined) and those without, there was no significant difference in the mean indirect bilirubin level between the two groups (232.5 ± 68.9 versus 226.8 ± 83.08 mmol/L, respectively) (*P* = 0.126). However, neonates with ABO incompatibility alone had a significantly higher mean indirect bilirubin level compared to those with other risk factors (234.9 ± 68.5 versus 225 ± 82.2 mmol/L, respectively) (*P* = 0.04). Yet, this finding was not significant for neonates with Rh incompatibility alone compared to other neonates (216.1 ± 74.1 versus 230.1 ± 77.6 mmol/L, respectively) (*P* = 0.31).

## 4. Discussion

In this study, ABO incompatibility was found to be the most common and a significant neonatal risk factor for indirect hyperbilirubinemia, accounting for 37.6% of all neonates. This result was supported by other studies in Saudi Arabia (31.6%), Egypt (12.9%), and Iran (16.9%) that also proved ABO incompatibility to be one of the commonest risk factors [3, 9, 11].

The second risk factor in the current study was G6PD deficiency, found in 32.5%. G6PD is one of the most common enzyme deficiencies globally; it affects approximately 200 million individuals [9]. It is an X-linked recessive disease which can cause indirect hyperbilirubinemia [10]. G6PD deficiency is considered a common problem in the Middle East, with a prevalence reaching up to 42% in neonates with indirect hyperbilirubinemia as shown in a previous study from Bahrain [8]. Moreover, a study from Saudi Arabia by Alkhotani et al. confirmed that 88.2% of G6PD-deficient patients in Makkah region developed neonatal indirect hyperbilirubinemia [9]. G6PD deficiency was frequently seen as a cause of neonatal indirect hyperbilirubinemia in different countries, including Saudi Arabia (10.5%), Egypt (10.1%), Iraq (10.5%), and Iran (6.3%) [7, 9, 11, 12].

In the current study, 21.8% of the neonates were premature. This is similar to a percentage reported by Shitran and Abed from Iraq, where 23.2% of their patients were premature [12]. Prematurity is a cause for indirect hyperbilirubinemia due to immature hepatic enzymes; this leads to impaired conjugation and increased levels of serum bilirubin [13].

Despite the fact that breastfeeding jaundice was a leading risk factor for developing neonatal indirect hyperbilirubinemia in countries like India and Ethiopia (48% and 30.7%), this was not the case in the current study, where only 7.6% had breastfeeding jaundice [14, 15]. This can be attributed to the possibility of higher percentages of breastfeeding in India and Ethiopia in comparison to Bahrain, where the introduction of formula milk is more affordable.

In the present study, 22% of newborns had no identifiable risk factors for developing indirect hyperbilirubinemia. This percentage is much lower than that reported by Pasha et al. from Iran, where 50.7% of newborns had no risk factors [3]. The presence of undetectable minor blood group incompatibilities might explain this variation.

In terms of maternal risk factors, in this study, 85.9% of neonates were born to mothers aged older than 25 years, making it the most common maternal factor for the development of indirect hyperbilirubinemia. Similarly, Sroufe et al. considered age of more than 25 years as a risk factor [16]. Girma also concluded that most mothers of patients with neonatal indirect hyperbilirubinemia were between 25 and 29 years [15]. However, maternal age cut point varied among studies. Two cross-sectional studies from Iran considered the age extremes, less than 18 and more than 35 years, as a risk factor [17, 18]. Moreover, Murekatete et al. reported that the majority of mothers with infants diagnosed with neonatal indirect hyperbilirubinemia were aged between 25 years and 35 years in Rwanda District Hospital [19].

When it comes to the mode of delivery, studies are conflicting. In the current study, 33.9% of the newborns had cesarean delivery as the second maternal risk factor. Moreover, Abd Elmoktader et al. found that cesarean delivery was more commonly seen in neonatal indirect hyperbilirubinemia cases [10]. Yet, other studies showed that vaginal delivery was a risk factor for developing indirect hyperbilirubinemia [12, 20]. Nonetheless, Tavakolizadeh et al. concluded that the mode of delivery has no association with the risk of developing neonatal indirect hyperbilirubinemia [17].

Upon comparison of neonates with risk factors and those without, males were significantly higher compared to females (51.7% versus 48.3%, respectively) (*P* = 0.008). Most studies agreed that male sex is a contributing factor for the development of neonatal indirect hyperbilirubinemia [16, 21]. Even in the United States of America, the percentage of males that developed indirect hyperbilirubinemia and kernicterus as a complication reached up to 67% of total infants, as reported by Johnson et al. [22]. These findings can explain why G6PD deficiency was a common risk factor, being an X-linked recessive disease, which is more in males. On the contrary, Garosi et al. from Iran found that female sex is associated with a severe form of indirect hyperbilirubinemia [23].

When comparing the nationality in relation to the risk factors of developing neonatal indirect hyperbilirubinemia, Bahraini nationals were found to be significantly higher, as 56.2% of Bahrainis had at least one risk factor compared to 21.5% of the non-Bahraini (*P* < 0.001). The high percentage of indirect hyperbilirubinemia in the Bahraini population may be attributed to the high prevalence of G6PD deficiency, as 46.1% of Bahraini nationals were G6PD deficient, while it affected 3.1% of the non-Bahraini population in this study (*P* < 0.0001).

Different types of therapies were used to manage indirect hyperbilirubinemia. Phototherapy is the primary treatment of indirect hyperbilirubinemia as it is a safe and effective method in lowering bilirubin levels [3]. In this study, it was used in 84.7% of the neonates at presentation. Exchange transfusion is a life-saving procedure especially for those with severe hyperbilirubinemia [3]. In the present study, ET was used in 14 (3.5%) neonates, 13 of which had multiple risk factors (3.2%). Moreover, IVIG was used only for those with risk factors (10.9%) (*P* = 0.001), and it was associated with higher bilirubin level compared to those who received other types of management. Al-Lawama et al. supported the safe administration of IVIG as an adjuvant treatment to phototherapy in neonates with isoimmune hemolytic diseases such as ABO incompatibility and Rh incompatibility, in which the levels of bilirubin were found to be higher [24]. This confirms the findings of our study where neonates with ABO incompatibility had a significantly higher mean indirect bilirubin compared to those with other risk factors (*P* = 0.04) and Rh incompatibility was detected as a risk factor for indirect hyperbilirubinemia in 27 (6.7%) newborns. Hemolytic disease of the newborn, which is also called erythroblastosis fetalis, is an important cause of indirect hyperbilirubinemia that might be severe when compared to other causes.

## 5. Study Limitations

This study was limited by its retrospective nature, which makes missing some patient's data expected. Moreover, some important laboratory tests such as the maternal indirect Coombs test, presence of anti-A antibodies, irregular antibodies, and others were not mentioned in this study. These tests are not routinely performed in our hospital. Yet, both maternal and neonatal blood groups, along with neonatal direct Coombs test, were tested for. Another limitation is that this study is a single-center study and of a local approach, and the generalization of the results might not be appropriate. However, the findings of this study can be beneficial to any institution providing care for neonates from neighbouring countries and worldwide. This study was also conducted in the same year of the coronavirus pandemic, which could be a reason behind late presentation or lost follow-up of some patients, as parents were hesitant to visit the hospital. Despite these limitations, this study focused on both maternal and neonatal risk factors for the development of indirect hyperbilirubinemia which was not fully covered by any previous studies from Bahrain. This study can help in antenatal and postnatal screening and can guide clinicians for better management of such cases to prevent the serious complications. The results of this study can also be used by future studies or metanalysis to further elaborate on this common pediatric condition.

## 6. Conclusions

ABO incompatibility, G6PD deficiency, and older maternal age (>25 years) were the commonest neonatal and maternal risk factors for developing neonatal indirect hyperbilirubinemia. Bahraini, male newborns, reticulocytosis, and IVIG use were associated with these factors. With the help of current screening methods, early detection of such risk factors is achievable. This is essential to prevent serious complications of neonatal indirect hyperbilirubinemia. Further studies are needed to evaluate the role of minor blood group incompatibility in the development and severity of neonatal indirect hyperbilirubinemia.

## Figures and Tables

**Table 1:** Demographic data of 404 neonates with indirect hyperbilirubinemia.

Demographic data	*n* (%)
Sex	
Male	209 (51.7)
Female	195 (48.3)
Nationality	
Bahraini	275 (68.1)
Non-Bahraini	129 (31.9)
Age at presentation (day), median (IQR^∗^)	2 (1-3)
Within 24 hours	150 (37.1)
1-5 days	230 (57)
>5 days	24 (5.9)
Gestational age (week), median (IQR)	38 (37-39)
Term	316 (78.2)
Preterm	88 (21.8)
Birth weight (kg), median (IQR)	3 (2.6-3.3)
Maternal age (year)	
<25	53 (14.1)
25 to 35	255 (60.6)
>35	102 (25.2)
Mode of delivery	
Vaginal	267 (66.1)
Cesarean	137 (33.9)
Feeding type (*n* = 157)	
Exclusive breast feeding	82 (52.2)
Exclusive formula milk	11 (7.0)
Mixed feeding	64 (40.8)
Newborn blood group	
O	158 (39.1)
B	123 (30.4)
A	108 (26.7)
AB	15 (3.7)
Maternal blood group	
O	232 (57.4)
B	81 (20.0)
A	77 (19.1)
AB	14 (3.5)
Newborn Rhesus factor	
Positive	384 (95)
Negative	20 (5)
Maternal Rhesus factor	
Positive	371 (91.8)
Negative	33 (8.2)
Positive direct Coombs test	97 (24)
Hospital stays (day), median (IQR)	3 (2-5)

Values are presented as numbers (%) for categorical variables and median (IQR) for continuous variables. IQR: interquartile range.

**Table 2:** Neonatal and maternal risk factors for the development of neonatal indirect hyperbilirubinemia (*n* = 404).

Neonatal risk factors	*n* (%)
ABO blood group incompatibility	152 (37.6)
Glucose-6-phosphate dehydrogenase deficiency (*n* = 400)	130 (32.5)
Prematurity	88 (21.8)
Polycythemia	30 (7.4)
Rhesus factor incompatibility	27 (6.7)
Breastfeeding jaundice (*n* = 157)	12 (7.6)
Cephalohematoma	9 (2.2)
Urinary tract infection (*n* = 91)	7 (7.7)
Sepsis (*n* = 168)	3 (1.8)
Congenital hypothyroidism (*n* = 160)	1 (0.63)
Maternal risk factors	n (%)
Maternal age > 25 years	357 (85.8)
Cesarean delivery	137 (33.9)
Maternal race (East Asian)	88 (21.8)
Gestational diabetes/diabetes mellitus	63 (15.6)
Maternal hypothyroidism	36 (8.9)
Maternal UTI	11 (2.7)
Maternal hyperthyroidism	2 (0.05)
No maternal risk factors determined	44 (10.9)

Values are presented as numbers (%). Patients might have more than one risk factor for neonatal indirect hyperbilirubinemia. UTI: urinary tract infection.

**Table 3:** Laboratory tests and radiological imaging of 404 neonates with indirect hyperbilirubinemia.

Investigations	Results	Normal values
Hemoglobin (g/dL), mean ± SD	16.9 ± 2.3	11-21.6
Hematocrit (%), mean ± SD	53.9 ± 7.8	<65%
Platelets count, median (IQR)	263 (202-337.8)	150 − 400 × 10^9^/L
White blood cell count, median (IQR)	10.7 (8.4-13.5)	3.6 − 9.6 × 10^9^/L
Reticulocytes (%), median (IQR)	3.6 (1.4-5.4)	0.5-1.5
Total serum bilirubin (*μ*mol/L), median (IQR)	236 (188-292)	<18
Indirect bilirubin (*μ*mol/L), median (IQR) (*n* = 404)	218 (174-270)	<18
Thyroid stimulating hormone (*μ*IU/mL), median (IQR) (*n* = 161)	3.5 (2.4-5.8)	0.52-16
Free thyroxin (T4) (*μ*g/dL), median (IQR) (*n* = 53)	27.5 (23.9-32.5)	5.9-21.5
Positive neonatal blood culture (*n* = 168)	3.0 (1.8)	—
Positive neonatal urine culture (*n* = 91)	7.0 (7.7)	—
Positive maternal urine culture (*n* = 263)	27 (10.3)	—
Positive maternal high vaginal swab (*n* = 107)	43 (40.2)	—
Positive HPLC (*n* = 393)	128 (32.6)	—
Skull ultrasound (*n* = 35)	1.0 (2.9)	—
Abdominal ultrasound (*n* = 18)	2.0 (11.1)	—

Values are presented as numbers (%), mean ± SD, and median (interquartile range). Fisher's exact test was used for categorical variables, while Student's *T* test and Mann–Whitney *U* test were used for continuous variables. SD: standard deviation; IQR: interquartile range; HPLC: high-performance liquid chromatography.

**Table 4:** Type of management for 404 neonates with indirect hyperbilirubinemia.

Management	*n* (%)	Indirect bilirubin level (*μ*mol/L)	*P* value
Phototherapy type	342 (84.7)		<0.0001
Single	182 (45.1)	199.8 ± 62.2	
Double	101 (25)	260.3 ± 68.8	
Triple	59 (14.6)	293.2 ± 72.5	
Exchange transfusion use			0.669
Yes	14 (3.5)	268.4 ± 142.79	
No	390 (96.5)	227.8 ± 73.9	
IVIG use			
Yes	44 (10.9)	258.7 ± 80.4	0.005
No	360 (89.1)	226.6 ± 76.4	

Values are presented as numbers (%) or mean ± SD. The Kruskal-Wallis test was used to compare different types of phototherapies, while the Mann–Whitney *U* test were used to compare neonates who received exchange transfusion and/or IVG with those who did not. *P* value < 0.05 was considered statistically significant. SD: standard deviation; IVIG: intravenous immunoglobulin.

**Table 5:** Comparison between neonates with risk factors of indirect hyperbilirubinemia versus those without.

Variable	Neonates with risk factors *n* (%) = 315 (88%)	Neonates without risk factors *n* (%) = 89 (22%)	*P* value (confidence interval)
Sex			0.008
Males	174 (43)	35 (8.7)	
Females	141 (35)	54 (13.3)	
Nationality			0.001
Bahraini	228 (56.5)	47 (11.6)	
Non-Bahraini	87 (21.5)	42 (10.4)	
Mode of delivery			0.835
Cesarean delivery	106 (26)	31 (7.7)	
Vaginal delivery	209 (52)	58 (14.3)	
WBC, mean ± SD	11.3 ± 4.67	12.1 ± 4.3	0.13 (-0.257 to 1.913)
Hematocrit, mean ± SD	53.8 ± 8.3	54.4 ± 5.5	0.39 (-0.841 to 2.111)
Hemoglobin, mean ± SD	16.8 ± 2.3	17.2 ± 2.1	0.08 (-0.067 to 1.000)
Platelets, mean ± SD	275 ± 97.3	272.3 ± 104.1	0.77 (-26.79 to 19.85)
Reticulocytes, mean ± SD	4.6 ± 2.6	3.7 ± 1.8	0.001 (-1.425 to -0.36)
Total bilirubin, mean ± SD	247.9 ± 82.3	246.5 ± 75.5	0.888 (-20.46 to 17.73)
Indirect bilirubin, mean ± SD	228.8 ± 78.9	230.5 ± 71.9	0.864 (-16.76 to 19.47)
Phototherapy use (*n* = 342)	271 (86)	71 (79.8)	0.817
Single	142 (52.4)	40 (56.3)	
Double	82 (30.3)	19 (26.8)	
Triple	47 (17.3)	12 (16.9)	
IVIG use	44 (10.9)	0.0 (0.0)	0.001
Exchange transfusion use	13 (3.2)	1.0 (0.2)	0.171

Values are presented as numbers (%) or mean ± SD. Fisher's exact test was used for categorical variables, while Student's *T* test and Mann–Whitney *U* test were used for continuous variables. *P* value < 0.05 was considered statistically significant. Confidence interval was set at 95%. WBC: white blood cells; SD: standard deviation; IVIG: intravenous immunoglobulin.

## Data Availability

The datasets generated during and/or analyzed during the current study are available from the corresponding author on reasonable request.
